# Fear of falling in age-related macular degeneration

**DOI:** 10.1186/1471-2415-14-10

**Published:** 2014-01-28

**Authors:** Suzanne W van Landingham, Robert W Massof, Emilie Chan, David S Friedman, Pradeep Y Ramulu

**Affiliations:** 1 Wilmer Eye Institute, Johns Hopkins University, 600 North Wolfe St. Maumenee B-110, Baltimore, MD 21287, USA

**Keywords:** Fear of falling, Falls, Age-related macular degeneration, Disability, Older adults, Visual acuity, vision loss, Physical function, Safety

## Abstract

**Background:**

Prior studies have shown age-related macular degeneration (AMD) to be associated with falls. The purpose of this study is to determine if (AMD) and AMD-related vision loss are associated with fear of falling, an important and distinct outcome.

**Methods:**

Sixty-five persons with AMD with evidence of vision loss in one or both eyes and 60 glaucoma suspects with normal vision completed the University of Illinois at Chicago Fear of Falling questionnaire. Responses were Rasch analyzed. Scores were expressed in logit units, with lower scores demonstrating lesser ability and greater fear of falling.

**Results:**

Compared to glaucoma suspect controls, AMD subjects had worse visual acuity (VA) (median better-eye VA = 20/48 vs. 20/24, p < 0.001) and worse contrast sensitivity (CS) (binocular CS = 1.9 vs. 1.5 log units, p < 0.001). AMD subjects were also older, more likely to be Caucasian, and less likely to be employed (p < 0.05 for all), but were similar with regards to other demographic and health measures. In multivariable models controlling for age, gender, body habitus, strength, and comorbid illnesses, AMD subjects reported greater fear of falling as compared to controls (β = -0.77 logits, 95% CI = -1.5 to -0.002, p = 0.045). In separate multivariable models, fear of falling increased with worse VA (β = -0.15 logits/1 line decrement, 95% CI = -0.28 to -0.03, p = 0.02) and CS (β = -0.20 logits/0.1 log unit decrement, 95% CI = -0.31 to -0.09, p = 0.001). Greater fear of falling was also associated with higher BMI, weaker grip, and more comorbid illnesses (p < 0.05 for all).

**Conclusions:**

AMD and AMD-related vision loss are associated with greater fear of falling in the elderly. Development, validation, and implementation of methods to address falls and fear of falling for individuals with vision loss from AMD are important goals for future work.

## Background

Falls are the leading cause of injury-related death in the elderly, and can lead to substantial morbidity [1]. Fear of falling can precede or result from falls, and has long been recognized as a distinct health outcome even more prevalent than falling itself [2]. It has been associated with decreased physical activity, reduction of social activity, lower quality of life, and nursing home admission [3-6].

Age-related macular degeneration (AMD) is a leading cause of vision loss in older adults, [7] and has been associated with falls [8-10]. Several other studies also suggest a dose-response relationship between falls and visual acuity (VA), with greater risk of falling observed in individuals with more severe vision loss [11,12]. AMD has also been associated with poor balance, difficulty with activities of daily living, smaller life-space, and poor quality of life, all of which may influence its relationship with falls and fear of falling [9,10,13,14].

No prior study has directly examined fear of falling in AMD patients, and only a few studies have examined the effect of vision loss on fear of falling. Fear of falling was assessed prospectively with a single question in the Beaver Dam Eye Study, which showed a 3-fold increase in incident fear of falling in individuals with a best-corrected binocular VA of 20/40 or worse [15]. Activity limitation due to fear of falling was assessed using a single question in patients with AMD, Fuchs, and glaucoma by Wang et al, and all three groups were more likely to report activity limitation due to fear of falling compared to controls [16]. Recently, an association has been demonstrated between glaucoma-related visual field loss and fear of falling using a validated questionnaire, [17] though it is not clear whether similar findings are likely to occur in patients with central visual disturbances and normal peripheral vision (as is typically encountered in AMD). Here, we use a previously validated questionnaire [17,18] in order to test the hypothesis that AMD is associated with fear of falling. We also examine the relationship between fear of falling and measures of vision loss in those with AMD.

## Methods

This cross-sectional study was approved by the Johns Hopkins Medicine Institutional Review Board and adhered to the tenets of the declaration of Helsinki. All subjects signed written informed consent and participated in the study between January 2010 and May 2012.

### Subjects

Subjects were recruited from among patients receiving care at the Wilmer Eye Institute of Johns Hopkins Hospital between July 2009 and June 2012. Subjects were between the ages of 60 and 80 years and met additional criteria qualifying them for either the AMD or glaucoma suspect (control) group as previously described [19].

Briefly, AMD subjects had a chart diagnosis of AMD with evidence of drusen, geographic atrophy, or choroidal neovascularization in both eyes. They were required to have a corrected VA of 20/32 or worse in both eyes, or 20/200 or worse in one eye regardless of the fellow eye’s VA. Glaucoma suspect controls all had a chart diagnosis of ocular hypertension or glaucoma suspect. They were required to have a corrected VA of 20/40 or better in each eye and recent Humphrey visual field testing demonstrating no significant visual field loss in either eye (mean deviation better than -5 dB in the worse-seeing eye and better than -3 dB in the better-seeing eye).

Exclusion criteria for both groups included ocular laser treatment or intraocular injections in the previous week, ocular surgery in the previous 2 months, non-ocular surgery or hospitalizations in the previous 2 weeks, and deterioration of the visual angle by greater than 50% within the past three months [20].

### Evaluation of fear of falling

Fear of falling was evaluated using the University of Illinois at Chicago Fear of Falling Questionnaire, which has been previously validated [17,18]. Questionnaires were administered orally to subjects during in-person interviews. Subjects were asked how worried they would be of falling if they were to perform each of 16 different activities. For example, the first item asks, “if you were to walk outside when icy, how worried are you of falling?” Three possible responses were accepted for each question: very worried, moderately worried/a little worried, or not at all worried.

### Evaluation of vision and covariates

Monocular VAs were measured using the Early Treatment Diabetic Retinopathy Study (ETDRS) chart transilluminated at 130 candelas/m^2^ and converted to the logarithm of the minimum angle of resolution (logMAR) [21]. VA of the better-seeing eye was used for further analysis. Binocular contrast sensitivity (CS) was measured using the Pelli-Robson chart at 1 meter with subjects wearing their habitual correction and converted into log units for analysis [22].

Both eyes were examined after pupillary dilation for significant nuclear sclerotic changes, cortical changes, posterior subcapsular cataract, or posterior capsular opacification (PCO) (in pseudophakic eyes) as previously described [23].

Demographic information collected included age, gender, race, employment status, years of education completed, living situation (if the subject lives with any other adults), and marital status, all by self-report. Height and weight were measured in clinic and used to calculate body mass index (BMI). Cognitive ability was assessed using the Mini Mental Status Exam for the Visually Impaired (MMSE-VI, scored from 1-22) [24]. Depressive symptoms were detected using the Geriatric Depression Scale Short Form, with subjects demonstrating 5 or more positive responses considered to have depressive symptoms [25]. Medical comorbidities were assessed using a standardized questionnaire and summarized as the number of comorbid conditions present [26]. Subjects were asked if a physician had ever diagnosed them with arthritis, broken or fractured hip, back problems, heart attack/myocardial infarction, angina/chest pain, congestive heart failure, peripheral vascular disease, hypertension, diabetes, emphysema, asthma, stroke, Parkinson’s, cancer (other than skin cancer), and vertigo/Meniere’s. Grip strength was assessed as a measure of overall frailty using a Jamar hand dynamometer (Sammons Preston, Inc, Bolingbrook, IL).

### Statistical analysis

Group differences for continuous variables were evaluated using the Student’s t-test. Chi-square analysis was used to assess differences in categorical variables.

Rasch analysis of the fear of falling questionnaire data was performed using Winsteps (Winsteps, Chicago, IL). Linear item measure scores for each task, representing task difficulty, and linear person measure scores for each person, representing the person’s ability or fear of falling, are expressed as log-odds units (logits) on a single scale (the “latent variable”). Higher item measure scores reflect more difficult activities, that is, activities that engender more fear of falling. Higher person measure scores reflect individuals less fearful of falling, or individuals who report little to no fear of falling when performing more difficult activities. This paper uses “fear of falling score” synonymously with “person measure score.” Differential item functioning (DIF) analysis was performed on the two study groups, AMD and glaucoma suspect controls, to investigate item bias. Item bias means that an item has different difficulty for the two groups and would suggest that items scale differently for the two groups. More detailed explanation of the Rasch methodology employed is available elsewhere [27].

The effects of AMD and vision loss on fear of falling person measure scores were first analyzed in age-adjusted models and subsequently in multivariable linear regression models with age, gender, BMI, grip strength, and number of comorbidities included as covariates (Stata 11.2, College Station, TX). Covariables were selected on the basis of significance in age-adjusted models. Race was not included in multivariable models because of the low numbers of African-Americans in the AMD group, and because several prior studies demonstrated no association between race and fear of falling, including recent work from the same institution [17,28-30]. Depression was not included due to the small number of subjects screening positive for depressive symptoms.

## Results

Sixty-five AMD subjects and 60 glaucoma suspect controls participated in the study. AMD and control subjects were similar with regards to most health and demographic characteristics including the presence of significant cataract/PCO, gender, education, living situation, body habitus, strength, comorbid illness, and cognitive ability (p > 0.05 for all) (Table 1). In comparison to control subjects, AMD subjects had worse VA (median logMAR of 0.38 versus 0.08 in the better-seeing eye, p < 0.001) and worse CS (median of 1.5 versus 1.9 log units, p < 0.001). The AMD group was also older (median age of 75.9 vs. 69.4, p < 0.001), had fewer African-Americans (1.5% vs. 20.0%, p = 0.001), and fewer employed persons (21.5% vs. 38.3%, p = 0.04) as compared to controls.

**Table 1:** Characteristics of study participants by group

	**Glaucoma suspect controls (n = 60)**	**AMD (n = 65)**	**p value**
**Vision**			
Median better-eye acuity, logMAR (IQR)	0.08 (0.0, 0.16)	0.38 (0.20, 0.06)	<0.001
Median better-eye acuity, Snellen	20/24	20/48	
Median binocular log contrast sensitivity (IQR)	1.9 (1.8, 2.0)	1.5 (1.4, 1.6)	<0.001
No. with cataract/PCO, either eye (%)	14 (23.2)	21 (32.3)	0.18
No. with cataract/PCO, both eyes (%)	6 (10.7)	11 (17.5)	0.29
**Demographics**			
Median age in years (IQR)	69.4 (65.2, 72.8)	75.9 (71.9, 78.3)	<0.001
No. African-American (%)	12 (20.0)	1 (1.5)	0.001
No. female (%)	37 (61.7)	38 (58.5)	0.72
Median education in years (IQR)	16.5 (14.0, 17.0)	16.0 (13.0, 17.0)	0.34
No. employed (%)	23 (38.3)	14 (21.5)	0.04
No. living alone (%)	11 (18.3)	14 (21.5)	0.65
**Health**			
Median body mass index in kg/m^2^ (IQR)	27.9 (23.8, 32.2)	27.4 (24.2, 32.5)	0.96
Median grip strength in kg (IQR)	26.3 (21.3, 32.3)	27.0 (21.3, 34.3)	0.66
Median no. comorbid illnesses (IQR)	2 (1, 3)	2 (1, 3)	0.94
No. with depressive symptoms (%)	3 (5.0)	3 (4.6)	0.92
Median MMSE-VI score, #/22 (IQR)	21 (20, 22)	21 (20, 22)	0.56

AMD = age-related macular degeneration, logMAR = logarithm of the minimum angle of resolution, IQR = interquartile range, PCO = Posterior Capsular Opacification, MMSE-VI = Mini-mental status examination for the visually impaired.

**Figure 1:**
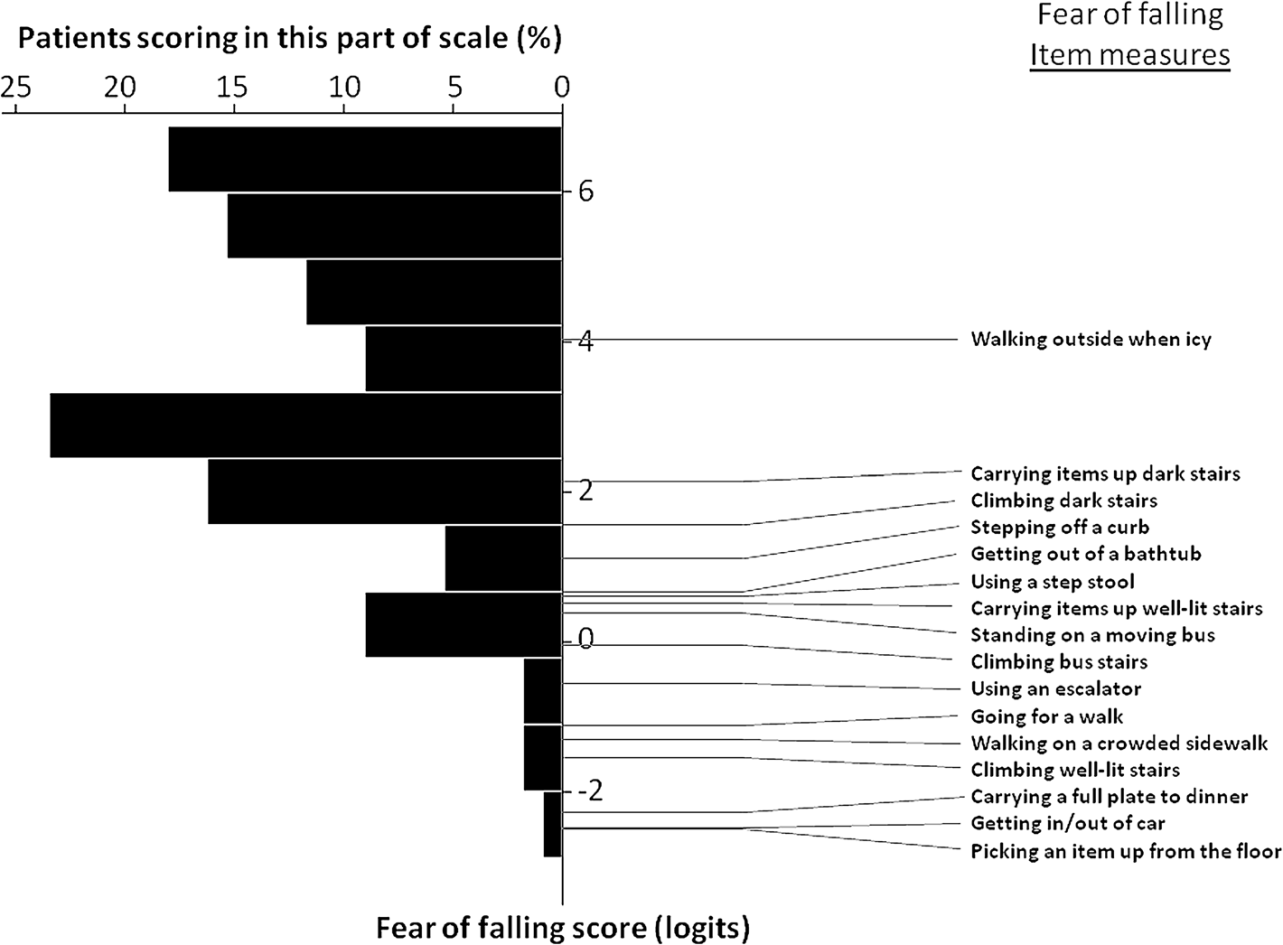
**Distribution of fear of falling item and person measures.** Study participants’ responses to the University of Illinois at Chicago Fear of Falling Questionnaire were analyzed using Rasch analysis. In this modified Wright plot, both item and person measures (fear of falling scores) are plotted along the latent variable (in logits, Y axis). A lower fear of falling score indicates greater fear of falling in the individual, whereas a lower item measure indicate less task difficulty.

A Rasch model was constructed using study subjects’ responses to the 16-item University of Illinois at Chicago Fear of Falling Questionnaire, aimed at quantifying each subject’s fear of falling as well as the difficulty level for each item along a latent variable. Item and person measure reliabilities were 0.77 and 0.97, respectively, indicating that 77% of the variance in the item measures (item difficulty level) and 97% of the variance in the person measures (fear of falling scores) were attributable to true differences in the items or people, and not to estimation error. The distribution of the fit statistics confirms the validity of the model for the study cohort. DIF analysis showed that all items scaled similarly for those with AMD and the controls, suggesting no substantial item bias. The distribution of item and person measure scores are shown in Figure 1.

**Figure 2:**
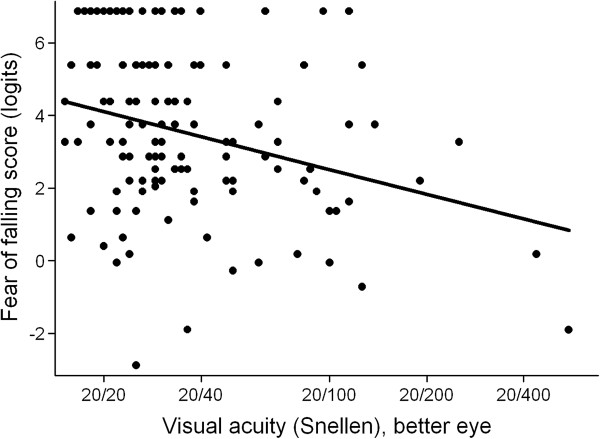
**Fear of falling scores as a function of visual acuity, unadjusted.** A lower fear of falling score indicates greater fear of falling, evidenced by fear with easier tasks. The scatter plot is shown with a line of best fit, y = 4.11 - 2.27x (p = 0.001). Worse visual acuity is associated with greater fear of falling.

**Figure 3:**
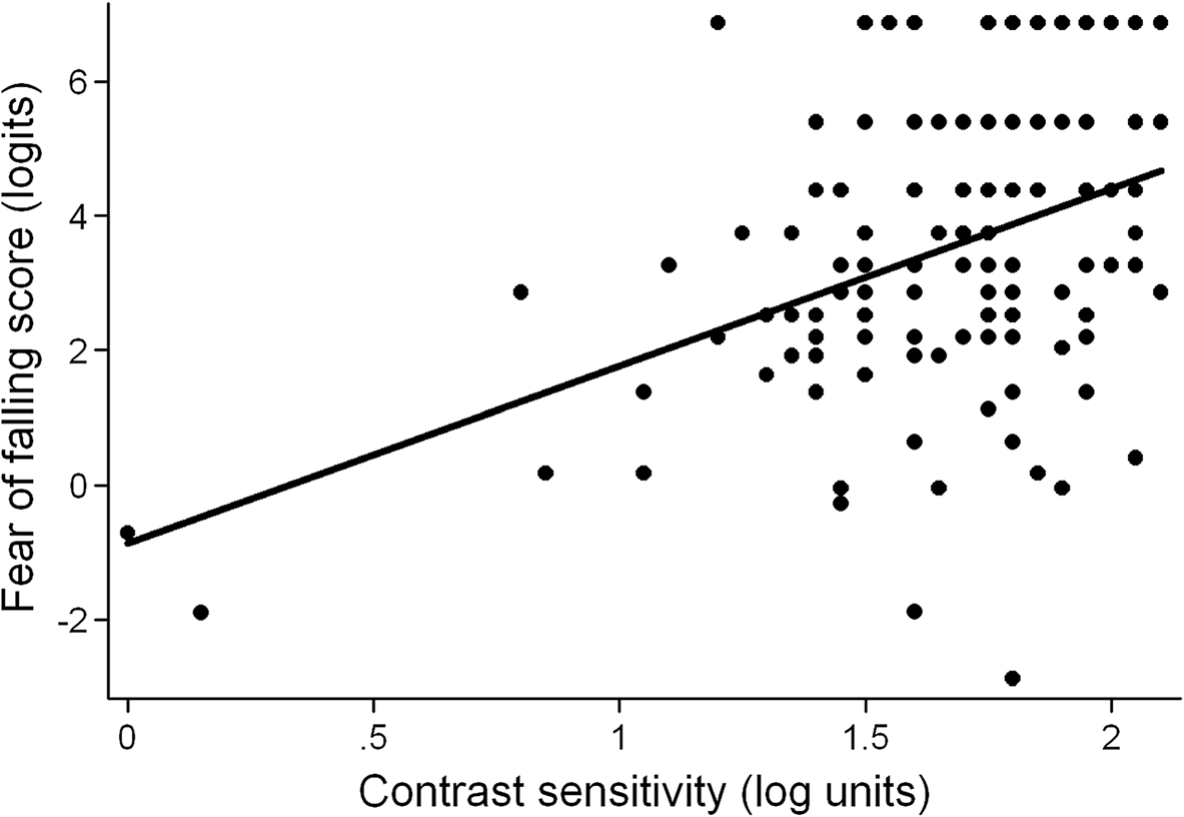
**Fear of falling scores as a function of contrast sensitivity, unadjusted.** A lower fear of falling score indicates greater fear of falling, evidenced by fear with easier tasks. Lower contrast sensitivity in log units indicates worse contrast sensitivity. The scatter plot is shown with a line of best fit, y = 2.65x – 0.87 (p < 0.001). Worse contrast sensitivity is associated with greater fear of falling.

AMD subjects as a whole did not demonstrate significantly greater fear of falling than controls in linear regression models adjusting only for age (β = -0.68 logits, 95% confidence interval [CI] = -1.5 to 0.28, p = 0.12) (Table 2), though greater fear of falling was associated with both worse VA (β = -0.2 logits/0.1 logMAR decrement, 95% CI = -0.33 to -0.06, p = 0.005) (Figure 2) and worse CS (β = -0.25 logits/0.1 log unit decrement in CS, 95% CI = -0.37 to -0.13, p < 0.001) (Figure 3). Other characteristics predicting fear of falling in age-adjusted models included female gender, employment, higher BMI, lower grip strength, comorbid illness, and depressive symptoms (p < 0.05 for all).

**Table 2:** Age-adjusted effect of vision, demographic, and health variables on fear of falling score (n = 125)

**Variable**	**Interval**	∆ fear of falling score logit units (95% CI)*	**Age-adjusted p value**
**Vision**			
Age-related macular degeneration	Present	-0.68 (-1.5 to 0.18)	0.12
Better-eye visual acuity	1 line worse†	-0.20 (-0.33 to -0.06)	0.01
Binocular log contrast sensitivity	0.1 log unit worse	-0.25 (-0.37 to -0.13)	<0.001
Cataract/PCO, either eye	Present, either eye	-0.47 (-1.3 to 0.40)	0.29
**Demographics**			
Age	5 years older	-0.08 (-0.15 to -0.01)	0.02
African-American race	vs. white	0.45 (-0.87 to 1.8)	0.50
Female	vs. male	-1.0 (-1.8 to -0.26)	0.01
Education	4 years more	0.49 (-0.17 to 1.1)	0.14
Employed	vs. unemployed	0.99 (0.07 to 1.9)	0.04
Lives alone	vs. lives with others	0.14 (-0.83 to 1.1)	1.00
**Health**			
Body mass index	1 kg/m^2^ higher	-0.08 (-0.14 to -0.02)	0.01
Grip strength	1 kg less force	-0.08 (-0.11 to -0.04)	<0.001
Comorbid illnesses	1 more illness	-0.38 (-0.62 to -0.14)	0.002
Depressive symptoms	Present	-3.2 (-4.9 to -1.4)	<0.001
MMSE-VI score	5 points lower	0.22 (-1.0 to 1.5)	0.72

PCO = Posterior Capsular Opacification, MMSE-VI = Mini-mental status examination for the visually impaired.

*Scores are derived from the Rasch analytic model. Higher scores indicate greater ability, or less fear of falling, so factors associated with a negative change in score are associated with greater fear of falling.

†Corresponds to 0.1 unit change in the logarithm of the minimum angle of resolution.

In multivariable linear regression models including age, gender, BMI, grip strength, and number of comorbid illnesses, AMD was associated with greater fear of falling (β = -0.77 logits, 95% CI = -1.5 to -0.002, p = 0.045) (Table 3). In separate multivariable models, greater fear of falling was associated with both better-eye VA (β = -0.15 logits/line decrease in VA, 95% CI = -0.28 to -0.03, p = 0.02) and binocular CS (β = -0.20 logits/0.1 log unit worse, 95% CI = -0.31 to -0.09, p = 0.001). Additional variables predictive of greater fear of falling in multivariable models included BMI, grip strength, and comorbid illness (p < 0.05 for all).

**Table 3:** Predictors of fear of falling, multivariable linear regression analysis (n = 125)

**Variable**	**Interval**	∆ fear of falling score logit units (95% CI)*	**p value**
**Vision‡**			
Age-related macular degeneration	Present	-0.77 (-1.5 to -0.002)	0.05
Better-eye visual acuity	1 line worse†	-0.15 (-0.28 to -0.03)	0.02
Binocular log contrast sensitivity	0.1 log unit worse	-0.20 (-0.31 to -0.09)	0.001
**Non-vision§**			
Age	5 years older	-0.03 (-0.09 to 0.04)	0.42
Female	vs. male	-0.18 (-1.2 to 0.80)	0.71
Body mass index	1 kg/m^2^ higher	-0.06 (-0.12 to 0.001)	0.04
Grip strength	1 kg less force	-0.07 (-0.13 to -0.02)	0.01
Comorbid illnesses	1 more illness	-0.28 (-0.50 to -0.06)	0.01

LogMAR = logarithm of the minimum angle of resolution, PCO = Posterior Capsular Opacification, MMSE-VI = Mini-mental status examination for the visually impaired.

*Scores are derived from the Rasch analytic model. Higher scores indicate greater ability, or less fear of falling, so factors associated with a negative change in score are associated with greater fear of falling.

†Corresponds to 0.1 logMAR change.

‡Coefficients for vision variables were derived from separate multivariable linear regression models including all non-visual covariates shown.

§Coefficients for non-vision variables were all derived from a single multivariable linear regression model including contrast sensitivity and all non-visual variables shown.

When CS and VA were included in a single model, CS remained a significant predictor of fear of falling (p = 0.02) whereas VA was no longer a significant predictor (p = 0.87).

## Discussion

AMD is associated with fear of falling in this cohort of older adults. The significance of this association is borderline, just meeting criteria for statistical significance in multivariable models, and not significant [p = 0.12] in models adjusting for age alone. VA and CS, however, are both strongly associated with greater fear of falling, indicating that fear of falling is greater in AMD patients with greater vision loss. Thus, the borderline statistical significance of group differences between AMD and controls may reflect the fact that some of our AMD group had a level of vision loss (mean better-eye VA = 20/48) insufficient to engender substantial fear of falling. Those with more advanced disease may be underrepresented in our group given that patients were gathered from a retina clinic, and not from a low vision clinic. Our study represents the first study to characterize fear of falling in AMD using a validated questionnaire [17].

Another notable finding is the fact that, in multivariable models including both VA and CS, only CS remained predictive of fear of falling. While both vision variables can be used to predict fear of falling, it is interesting that CS is the better predictor, as it is not routinely assessed outside of low vision practices. Similarly, Wang et al found CS to be the measure of visual function most important to describing the impact of eye disease on activity limitation due to fear of falling [16]. CS has been shown to correlate strongly with balance, falls, injuries, and other functional outcomes in previous studies, perhaps arguing for more frequent clinical use of CS [11,31-34].

The results of the present study should be considered in conjunction with our recent finding of an association between binocular visual field loss from glaucoma and fear of falling [17]. These two studies reveal links between fear of falling and vision loss from the two most common causes of irreversible vision loss in the United States, AMD and glaucoma [35]. This is particularly interesting given the different ways in which these two diseases cause vision loss, with glaucoma first causing loss of peripheral vision and AMD first causing loss of central vision and visual acuity. It is possible that substantial vision loss of any cause could engender fear of falling.

Falls have been related to AMD and AMD-related vision loss previously. In one prospective study that used monthly phone calls for falls assessment, the age-adjusted incident rate ratio for injurious falls was 1.8 for women with neovascular AMD compared to those without [8]. Another prospective study using diaries and monthly follow-up to track falls found that among AMD patients, those with worse VA and CS were more likely to report falling [11]. Several other studies have also linked AMD to falls but were limited because they assessed falls retrospectively [9,30], and retrospective fall results correlate poorly with prospective falls evaluation [31].

No prior study has directly examined fear of falling in AMD patients, and only a few studies have examined the effect of vision loss on fear of falling. Fear of falling was assessed prospectively with a single question in the Beaver Dam Eye Study, which showed a 3-fold increase in incident fear of falling in individuals with a best-corrected binocular VA of 20/40 or worse, though specific eye diseases were not identified [15]. Wang et al assessed activity limitation due to fear of falling in patients with AMD, Fuchs, and glaucoma using a single dichotomous question, and found all three groups to be at increased risk for reporting activity limitation due to fear of falling compared to controls [16].

These interrelationships between AMD, falls, and fear of falling are likely multifaceted. One possible mechanism is that AMD-related vision loss leads to balance problems, [11,13] which lead to falls and fear of falling, then to decreased walking and physical activity, and subsequent depression/anxiety, social isolation, frailty, injury, and death. Falls may result in fear of falling, but fear of falling may also precede falls or exist in the absence of falls. One study has even identified self-reported decreased life space and limited activities due to fear of falling as apparent mediators of the relationship between eye disease and depression, indicating that fear of falling is a key component of decreased quality of life in persons with eye disease [36].

Given that people with AMD are at higher risk for fear of falling, and that fear of falling represents a risk factor for both falls and decreased quality of life, these findings emphasize the importance of addressing falls and fear of falling in this group. There is preliminary evidence that balance training can improve balance in low vision groups, though its impact on falls and fear of falling is unclear [37]. It is likely that orientation and mobility training has an impact on falls and fear of falling, but this has yet to be formally studied. Further study is needed to determine the best way to address falls and fear of falling in low vision rehabilitation, with an aim of reducing injurious falls, increasing or maintaining life-space and social functioning, and improving overall quality of life.

Our study also found fear of falling to be associated with body habitus, strength, and comorbid illnesses, which confirms findings from previous studies [2,17,38]. Other studies have found associations of fear of falling with older age and female gender, as well. The lack of association with age observed in the present study could be because we only included subjects in a fairly narrow age range.

One strength of our study is that we analyzed fear of falling using a previously validated questionnaire and a Rasch analytic model [18]. The questionnaire used here was originally validated for a general elderly population, but functioned well in both the present study as well as in a previously described group of glaucoma patients, demonstrating good person and item measure [17,18].

Our study has several limitations. Although we performed study procedures on the same day as a clinical visit wherever possible, participation in the study required extra time in or an extra trip to the clinic, which may have been more burdensome to those with lower physical ability (and greater fear of falling) and discouraged their participation. If present, however, this bias would likely result in an underestimate of the relationship between fear of falling and vision loss. Another potential limitation is that our control subjects are glaucoma suspects rather than true ‘normals’ not under ophthalmologic care. The visual characteristics of these suspects with regards to acuity, visual field mean deviation and contrast sensitivity were highly similar to a population-based sample of older adults from Maryland [39]. This group was chosen as a control group instead of patients’ friends or spouses as friends and spouses are likely to present for study recruitment only if they have relatively good mobility, which could result in a supranormal control group and bias our study towards a positive finding.

## Conclusions

In conclusion, AMD is associated with greater fear of falling, and fear of falling is greater in patients with worse visual acuity and contrast sensitivity. The importance of fear of falling is well documented, and these new findings indicate that it may be a key factor linking AMD to decreased quality of life and disability. Development, validation, and implementation of methods to address falls and fear of falling for individuals with vision loss from AMD and other causes of vision loss is an important goal for future work, and may be a key component to preventing or moderating disability caused by AMD-related vision loss.

## Abbreviations

AMD: Age-related macular degeneration; VA: Visual acuity; CS: Contrast sensitivity; CI: Confidence interval; BMI: Body mass index; ETDRS: Early Treatment Diabetic Retinopathy Study; logMAR: Logarithm of the minimum angle of resolution; PCO: Posterior capsular opacification; MMSE-VI: Mini mental status exam for the visually impaired; DIF: Differential item functioning.

## Competing interests

The authors declare that they have no competing interests.

## Authors’ contributions

SV collected data, participated in data analysis, and drafted the manuscript. RM participated in data analysis. EC collected data. DF contributed to study design and data analysis. PY contributed to study design, lead the study team, and participated in data analysis. All authors helped to revise the manuscript as well as read and approved the final manuscript.

## Pre-publication history

The pre-publication history for this paper can be accessed here:

http://www.biomedcentral.com/1471-2415/14/10/prepub
